# DNA methylation profile dynamics of tissue-dependent and differentially methylated regions during mouse brain development

**DOI:** 10.1186/1471-2164-14-82

**Published:** 2013-02-06

**Authors:** Keiji Hirabayashi, Kunio Shiota, Shintaro Yagi

**Affiliations:** 1 Laboratory of Cellular Biochemistry, Department of Animal Resource Sciences/Veterinary Medical Sciences, The University of Tokyo, Yayoi 1-1-1, Bunkyo-ku, Tokyo 113-8657, Japan

**Keywords:** DNA methylation, Tissue-dependent and differentially methylated region, Neural progenitor cells

## Abstract

**Background:**

Tissues and their component cells have unique DNA methylation profiles comprising DNA methylation patterns of tissue-dependent and differentially methylated regions (T-DMRs). Previous studies reported that DNA methylation plays crucial roles in cell differentiation and development. Here, we investigated the genome-wide DNA methylation profiles of mouse neural progenitors derived from different developmental stages using HpyCH4IV, a methylation-sensitive restriction enzyme that recognizes ACGT residues, which are uniformly distributed across the genome.

**Results:**

Using a microarray-based genome-wide DNA methylation analysis system focusing on 8.5-kb regions around transcription start sites (TSSs), we analyzed the DNA methylation profiles of mouse neurospheres derived from telencephalons at embryonic days 11.5 (E11.5NSph) and 14.5 (E14.5NSph) and the adult brain (AdBr). We identified T-DMRs with different DNA methylation statuses between E11.5NSph and E14.5NSph at genes involved in neural development and/or associated with neurological disorders in humans, such as *Dclk1*, *Nrcam*, *Nfia*, and *Ntng1*. These T-DMRs were located not only within 2 kb but also distal (several kbs) from the TSSs, and those hypomethylated in E11.5NSph tended to be in CpG island (CGI-) associated genes. Most T-DMRs that were hypomethylated in neurospheres were also hypomethylated in the AdBr. Interestingly, among the T-DMRs hypomethylated in the progenitors, there were T-DMRs that were hypermethylated in the AdBr. Although certain genes, including *Ntng1*, had hypermethylated T-DMRs 5^′^ upstream, we identified hypomethylated T-DMRs in the AdBr, 3^′^ downstream from their TSSs. This observation could explain why *Ntng1* was highly expressed in the AdBr despite upstream hypermethylation.

**Conclusion:**

Mouse adult brain DNA methylation and gene expression profiles could be attributed to developmental dynamics of T-DMRs in neural-related genes.

## Background

The adult mouse brain consists of various kinds of cells that sequentially appear as neurons, astrocytes, and oligodendrocytes from late gestation through the neonatal period. Distinctive neural progenitor cells (NPCs) that exhibit different differentiation potentials to neurons and glial cells are generated during mid-to-late gestation [1-3]. This process is controlled by signaling pathways composed of transcription factors; dysfunction in genes encoding these factors is known to result in brain malformation [4-6].

Epigenetic systems underlie the network of tissue- and developmental stage-specific transcription factors and their targets [7]. Major players in epigenetic systems are DNA methylation and histone modifications, which occur on nucleosomes and affect chromosomal activity by changing nucleosome architecture. Tissue-dependent and differentially methylated regions (T-DMRs) are found throughout the genome and influence tissue-specific gene expression. T-DMRs have been found 3^′^ downstream of transcription start sites (TSSs) in addition to in 5^′^-upstream promoter regions. A distinct combination of DNA methylation patterns at T-DMRs determines cellular identity during development [8-13], thus illustrating that DNA methylation profiles are unique to individual cells or tissue types [7,14,15].

Genome-wide DNA methylation analyses focusing on proximal promoter regions in embryonic stem cell-derived NPCs and NPCs committed to astrocytes indicate the importance of DNA methylation in the commitment process and differentiation potential of NPCs [16-19]. These reports indicated that the majority of DNA methylation changes occur at low-CpG density promoters, suggesting sequence preferences in DNA methylation targets during neural differentiation [18]. However, T-DMRs are observed at high-CpG density promoters, such as those containing CpG islands (CGIs), and are tissue-dependently methylated in the adult brain (AdBr) [7,20].

In this study, we performed a comparative analysis of DNA methylation status in NPCs derived from mid- and late-gestation mouse embryo. Using microarray-based, genome-wide DNA methylation profiling [7], we identified T-DMRs in dozens of genes, and we illustrate dynamic DNA methylation statuses for dozens of T-DMRs, which are reflected in the DNA methylation profile of the AdBr.

## Results

### Distinct DNA methylation profiles in NPCs with different fates

**Figure 1:**
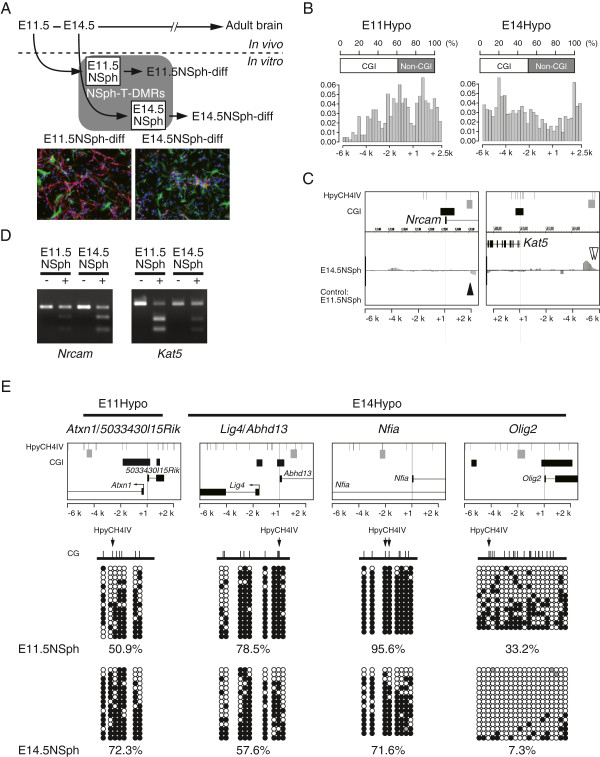
**Distinct DNA methylation profile between E11.5NSph and E14.5NSph.** (**A**) Schematic of the analysis in this study. E11.5NSph and E14.5NSph were cultured from telencephalons of E11.5 and E14.5 mouse embryos and used as models of NPCs. Comparative analysis of D-REAM data was performed to identify NSph-T-DMRs. Immunocytochemical analysis of differentiated NSphs (E11.5NSph-diff and E14.5NSph-diff) was conducted using antibodies against βIII-tubulin (TUBB3) and glial fibrillary acidic protein (GFAP). TUBB3-positive and GFAP-positive cells are indicated in red and green, respectively, and DAPI-stained nuclei are indicated in blue. (**B**) Distinct characteristics of E11Hypo-T-DMRs and E14Hypo-T-DMRs. The proportion of CGI genes (upper bar charts) and the distributions of NSph-T-DMRs to TSSs (lower panels) are displayed. E11Hypo-T-DMRs (left) and E14Hypo-T-DMRs (right) were mapped in 208 and 604 genes, respectively. The y-axis represents the proportions of each fraction to the whole as 1. The width of the histogram is 250 bp. (**C**) Integrated Genome Browser (IGB) images of *Nrcam* and *Kat5* gene loci (Ensembl Transcripts) showing comparative MATscores of E14.5NSph to E11.5NSph. Filled and open arrowheads indicate E11Hypo-T-DMRs and E14Hypo-T-DMRs, respectively. Regions analyzed by COBRA (D) are represented by gray rectangles. (**D**) COBRA representing DNA methylation status of HpyCH4IV sites in *Nrcam* and *Kat5* gene regions. Bisulfite PCR products using genomic DNA from E11.5NSph and E14.5NSph were not treated (−) or treated with HpyCH4IV (+) and electrophoresed. (**E**) DNA methylation status of the indicated regions located in 4 disease-associated genes (gray rectangles of the upper panels) was analyzed by bisulfite sequencing. Each open, filled, and gray circle represents unmethylated, methylated CpG, and CpG with an undetermined methylation state, respectively. Percentages of methylated CpGs are indicated.

To explore DNA methylation profiles of mouse NPCs, we compared neurospheres (NSph) derived from telencephalons at embryonic days 11.5 (E11.5NSph) and 14.5 (E14.5NSph) by T-DMR profiling with restriction tag-mediated amplification (D-REAM) with mouse promoter tiling arrays covering from 6 kb upstream to 2.5 kb downstream of 30,140 gene TSSs (Ensembl Transcript IDs) [7]. The distinctive cell fates of E11.5NSph and E14.5NSph were indicated by biased expression of marker genes for neural and oligodendrocyte progenitor in the undifferentiated NSph, and those of neuronal and glial marker genes in the differentiated ones, respectively (Figure 1A and Additional file 1: Figure S1). We screened genomic regions that exhibited differential MATscores [7,21] between NSphs, which indicate differential methylation status, and obtained a total of 1,403 NSph-T-DMRs consisting of 380 E11Hypo-T-DMRs and 1,023 E14Hypo-T-DMRs, which were hyper- and hypomethylated, respectively, in E14.5NSph compared to E11.5NSph.

The localization patterns along the genome were distinct between E11Hypo-T-DMRs and E14Hypo-T-DMRs. The former exhibited bimodal distributions within 2.5 kb from TSSs and biased to the CGI genes, which contain CGIs around TSSs [7]; the latter were located 6 kb to 2 kb upstream of TSSs without any promoter type bias (Figure 1B). These findings are noteworthy because they indicate that methylation changes occur in regions around high-CpG promoters. We analyzed E11Hypo-T-DMR 3^′^ downstream from the *Nrcam* TSS, and E14Hypo-T-DMR 5^′^ upstream from the *Kat5* TSS. Combined bisulfite restriction analysis (COBRA) of these T-DMRs indicated differential DNA methylation status as indicated by D-REAM (Figure 1C and 1D).

Among the genes with NSph-T-DMRs, we identified human gene orthologs involved in neurological diseases, such as spinocerebellar ataxia type 1 (*ATXN1* and *KAT5*), schizophrenia (*BLOC1S1*, *NTNG1*, and *OLIG2*), autism (*NRCAM*), and brain malformation syndrome (*LIG4* and *NFIA*) (Additional file 2: Table S1). We performed bisulfite sequencing of 1 E11Hypo-T-DMR (*Atxn1*) and 3 E14Hypo-T-DMRs (*Lig4*, *Nfia*, and *Olig2*) that were located at various relative positions from the TSSs, including one associated with an alternative TSS and those facing their TSS in proximal regions. The results showed clear differences in the DNA methylation statuses of CpG sites around the HpyCH4IV sites between the 2 types of NSphs (Figure 1E). These data indicate that methylation changes occurred in a subpopulation of NSphs in a gene-dependent manner.

### Association of NSph-T-DMRs with neural development and function

We performed gene ontology (GO) analysis to characterize genes with the NSph-T-DMRs. The neural-related GO term “central nervous system development” was enriched in genes with E11Hypo-T-DMRs but was not enriched in those with E14Hypo-T-DMRs (Tables 1 and 2). Genes with E11Hypo-T-DMRs included those for neuronal differentiation and functions: specification of retinal amacrine neurons (*Barhl2*), axon outgrowth (*Dclk1*), inhibition of oligodendrocyte differentiation (*Id2*), and axon guidance (*B3gnt2* and *Nrcam*). Among genes with E14Hypo-T-DMRs, we found those involved in astrocyte and/or oligodendrocyte development, such as *Nfia* and *Olig2*. Both E11Hypo- and E14Hypo-T-DMR genes included those involved in cell fate commitment (*Barhl2*, *Olig2*, and *Cdon*) and brain morphogenesis (*Tcfap2a*, *Fezf1*, *Cer1*, and *Cdon*). A search of the OMIM (Online Mendelian Inheritance in Man) database indicated that genes with E14Hypo-T-DMRs that encode membrane-associated proteins (*Accn1*, *Scg5*, and *Slc15a2*) are expressed in the AdBr (Table 2). Thus, developmental stage-specific methylation and demethylation at the T-DMRs in genes related to neuronal and glial development occurred during neural cell fate determination.

**Table 1:** Annotation analysis of genes with NSph-T-DMRs

**Genes with E11Hypo-T-DMRs**		
**Category**	**Term**	**P value**
BP	Central nervous system development	1.01E-02
MF	Cyclin-dependent protein kinase inhibitor activity	1.57E-03
KEGG	Propanoate metabolism	1.40E-03
**Genes with E14Hypo-T-DMRs**		
BP	Positive regulation of developmental process	8.08E-03
CC	Peroxisome	7.43E-03
MF	Transmembrane transporter activity	1.41E-02
KEGG	Retinol metabolism	2.80E-03

BP, MF, KEGG, and CC indicate biological process, molecular function, KEGG pathway, and cellular component, respectively.

**Table 2:** Genes with NSph-T-DMRs annotated for neural development and functions

**NSph-T-DMR**	**Gene**	**Description**	**GO terms related to development and neural functions**	**OMIM**
E11Hypo	*Atxn1*	Ataxin 1	Transmission of nerve impulse	601556
E11Hypo	*B3gnt2*	UDP-GlcNAc:betaGal beta-1,3-N-acetylglucosaminyltransferase 2	Ax, ND, sensory perception	605581
E11Hypo	*Barhl2*	BarH-like 2 (Drosophila)	Ax, CC, ND	605212
E11Hypo	*Cdkn2a*	Cyclin-dependent kinase inhibitor 2A		600160
E11Hypo	*Dclk1*	Doublecortin-like kinase 1	Ax, ND	604742
E11Hypo	*Id2*	Inhibitor of DNA binding 2		600386
E11Hypo	*Nrcam*	Neuron-glia-CAM-related cell adhesion molecule	Ax, ND, transmission of nerve impulse	601581
E11Hypo	*Tcfap2a*	Transcription factor AP-2, alpha	neural tube closure	107580
E11Hypo/E14Hypo	*Cer1*	Cerberus 1 homolog (Xenopus laevis)		603777
E14Hypo	*Accn1*	Amiloride-sensitive cation channel 1, neuronal (degenerin)	Sensory perception	601784
E14Hypo	*Aldh1a2*	Aldehyde dehydrogenase family 1, subfamily A2	ND	603687
E14Hypo	*Atp11a*	ATPase, class VI, type 11A		605868
E14Hypo	*Bloc1s1*	Biogenesis of lysosome-related organelles complex-1, subunit 1		601444
E14Hypo	*Cdon*	Cell adhesion molecule-related/down-regulated by oncogenes	CC	608707
E14Hypo	*Dph5*	DPH5 homolog (S. cerevisiae)		611075
E14Hypo	*Emid1*	EMI domain containing 1		608926
E14Hypo	*Fezf1*	Fez family zinc finger 1	Ax, ND	613301
E14Hypo	*Kat5*	K(lysine) acetyltransferase 5		601409
E14Hypo	*Lig4*	Ligase IV, DNA, ATP-dependent	Neuron apoptosis	601837
E14Hypo	*Mcf2l*	Mcf.2 transforming sequence-like		609499
E14Hypo	*Nfia*	Nuclear factor I/A		600727
E14Hypo	*Ntng1*	Netrin G1	Ax, ND	608818
E14Hypo	*Olig2*	Oligodendrocyte transcription factor 2	CC, ND, gliogenesis, transmission of nerve impulse	606386
E14Hypo	*Scg5*	Secretogranin V	Neuropeptide signaling pathway	173120
E14Hypo	*Slc15a2*	Solute carrier family 15 (H+/peptide transporter), member 2		602339
E14Hypo	*Wasl*	Wiskott-Aldrich syndrome-like (human)		605056

Ax, axonogenesis; CC, cell fate commitment; ND, neuron differentiation.

### DNA methylation profile of NSph-T-DMRs in the AdBr

**Figure 2:**
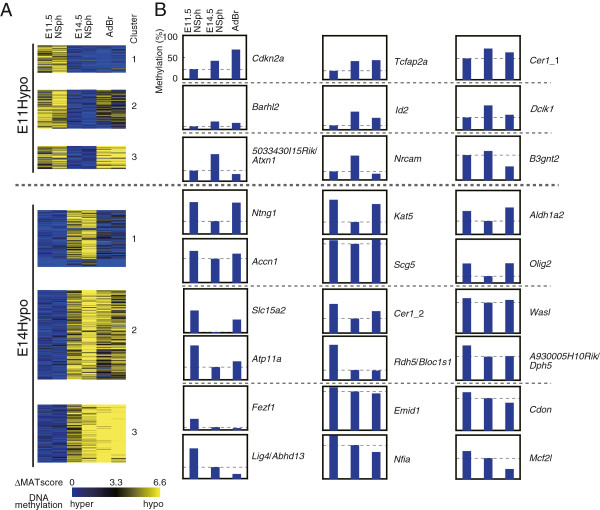
**Stage-specific DNA methylation profile of NSph-T-DMRs in NPCs.** (**A**) K-means clustering of the regions corresponding to NSph-T-DMRs by Pearson’s correlations of their MATscores. The delta MATscores (ΔMATscores) were obtained by comparing duplicate D-REAM data of the AdBr, and E11.5NSph or E14.5NSph, with those of E14.5NSph (for E11Hypo) and E11.5NSph (for E14Hypo), respectively, and were displayed as heatmaps. The range of ΔMATscores, which indicate differentially methylated status at the loci, is indicated at the bottom of the panels. (**B**) Band intensities of electrophoresis images of COBRA were analyzed densitometrically, and methylation percentages were calculated and plotted. Dotted lines indicate methylation percentages of E11.5NSph for E11Hypo-T-DMRs and those of E14.5NSph for E14Hypo-T-DMRs.

We compared D-REAM data between NSphs and the AdBr and used K-means clustering to classify E11hypo- and E14hypo-T-DMRs into 3 clusters. In the AdBr, most E11Hypo-T-DMRs and E14Hypo-T-DMRs exhibited hypomethylation (clusters 2 and 3) (Figure 2A). Although the degrees of differences varied among genes, COBRA of NSph-T-DMRs indicated hypomethylated status at some loci as clusters 2 and 3 (e.g., *Dclk1* and *B3gnt2* for E11Hypo-TDMR, and *Rdh5*/*Bloc1s1* and *Mcf2l* for E14Hypo-T-DMRs), and hypermethylated status at other loci as cluster 1 (e.g., *Cdkn2a* and *Ntng1* for E11Hypo- and E14Hypo-T-DMRs, respectively) in the AdBr (Figure 2B and Additional files 3 and 4: Tables S2 and S3).

**Figure 3:**
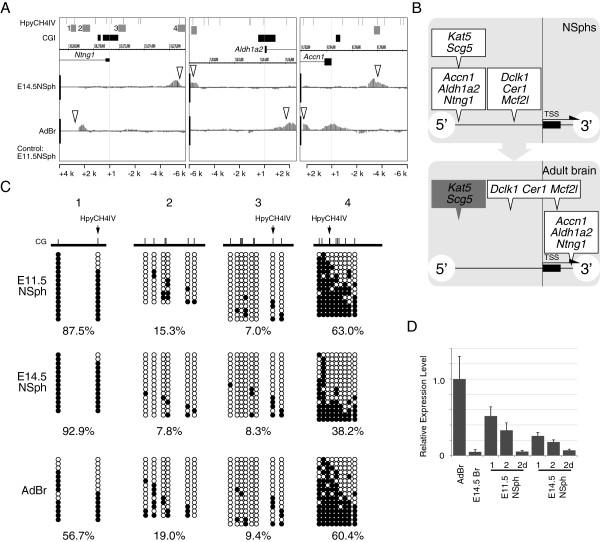
**A shift of the hypomethylated region from 5**^′^**upstream to 3**^′^**downstream in adulthood.** (**A**) IGB images of the 3 genes with 5^′^-upstream region E14Hypo-T-DMRs. Comparative MATscores of E14.5NSph and the AdBr to E11.5NSph as the control were plotted and displayed from 6 kb upstream to 4 kb (*Ntng1*) or 2.5 kb downstream of the TSS. Open arrowheads indicate hypomethylated T-DMRs. Regions analyzed by bisulfite sequencing (C) or COBRA are indicated by gray rectangles. (**B**) Relative positions of hypomethylated T-DMRs to TSS in NSphs (upper panel) and the adult brain (lower panel) are illustrated. (**C**) DNA methylation status of *Ntng1* in E11.5NSph, E14.5NSph, and the AdBr was analyzed by bisulfite sequencing. Each open and filled circle represents unmethylated and methylated CpG, respectively. Percentages of methylated CpGs are indicated. (**D**) Expression of *Ntng1* was analyzed by Q-RT-PCR using total RNA from undifferentiated (1, 2) and differentiated NSph (2d), E14.5 telencephalon (E14.5Br), and the AdBr. The levels of expression, which were normalized with those of internal control (*Actb*), are indicated by the ratios to the average of AdBr. Error bars indicate SE of the triplicated PCR.

Among genes with cluster-1 E14Hypo-T-DMRs, we unexpectedly found that T-DMR hypermethylation was associated with higher gene expression in the brain (described later). To address this issue, we further investigated the DNA methylation status of other HpyCH4IV sites in these genes using AdBr D-REAM data and found AdBr-specific hypomethylated T-DMRs 3^′^ downstream of their TSSs in *Ntng1*, *Aldh1a2*, and *Accn1* (Figure 3A). It is noteworthy that all these T-DMRs were located within few kb from CGIs.

The positional changes of hypomethylated T-DMRs in a specific genomic region are summarized in Figure 3B. Bisulfite sequencing analysis of T-DMRs in the *Ntng1* gene indicated hypermethylation of E14Hypo-T-DMRs at the 5^′^-upstream region and hypomethylation at the 3^′^ downstream of the TSS in the AdBr with unmethylated neighboring regions in all samples (Figure 3C). Quantitative reverse-transcription polymerase chain reaction (Q-RT-PCR) data indicated negative correlation between hypomethylation at distal T-DMR (region 4) in undifferentiated NSphs, and an association of gene expression in AdBr with hypomethylation of the T-DMR 3^′^ downstream of the CGI (Figure 3D). These results highlight functions associated with developmental stage-dependent multiple T-DMRs in a gene region.

## Discussion

Comparing NSphs with different cell fates enabled the identification of numerous T-DMRs in genes at different relative positions from TSSs. DNA methylation and demethylation occurred in a developmental stage-dependent manner, and changes in DNA methylation at these T-DMRs resulted in variable methylation in AdBr cells that shifted the DNA methylation profile as a whole. The hypomethylated status of most NSph-T-DMRs was reflected in the DNA methylation profile of the AdBr to different degrees in a locus-specific manner. The previous genome-wide methylation analyses of NPCs [16-18] emphasized preexisting epigenetic marks, such as bivalent histone modifications on poised genes involved in early differentiation processes and demethylated promoters of astrocyte-specific genes in progenitor cells preceding expression in differentiated cells. DNA methylation status in NSphs and gene expression in the AdBr have led to the hypothesis that a considerable number of T-DMRs identified in this study are epigenetically marked prior to gene expression. The developmental-stage specific DNA methylation marks could be useful for identify and evaluation of NPCs established from not only fetus but also stem cells as pluripotent stem cells and those from adult tissues.

We observed developmental position changes such as 5^′^ distal hypomethylated T-DMRs in the NSphs and hypomethylated T-DMR marks 3^′^ proximal downstream of TSSs in the fully developed brain. These T-DMRs were often located around CGIs, which is in contrast to a previous genome-wide analysis of NPCs indicating biased DNA methylation changes to low-CpG promoters [17,18]. T-DMRs found in the *Ntng1* locus could be classified into the previously described class of T-DMRs downstream of TSSs of CGI genes, in which hypomethylation was well correlated with gene expression [7,20]. T-DMRs have been identified at the edges of CGIs [22], and DMRs around CGIs, named as CpG island shores, are identified in not only normal tissues but also cancer cells [23]. The biased distribution of E14Hypo-T-DMRs to the relatively 5^′^-distal positions from TSSs suggested that hypomethylation of these 5^′^-distal T-DMRs in the progenitor cells are epigenetic marks that lead to expression in differentiated cells, which exhibit hypomethylation of T-DMRs at 3^′^ downstream of TSS.

Systematic biases are inevitable for any genome-wide DNA methylation analysis: both Microarray-based Integrated Analysis of Methylation by Isoschizomers (MIAMI) [16] and Reduced Representation Bisulfite Sequencing (RRBS) [18], methods used in the previous epigenomic study in NPCs, are inevitably focusing on CGIs because of the biased distribution of MspI recognition sites that they uses for enrichment of fragments [24]; Methylated DNA immunoprecipitation (MeDIP) [17], is known to have bias to high density CpG promoter [25]. Approximately 50% of promoters are associated with CGIs. D-REAM also has [7,25]. Only limited numbers of genes are coincided to be predicted to have DMRs in NSph: *Gfap*, which have been shown to have DMR hypomethylated in E14.5NSph [18], was not included in our gene list because of lacking HpyCH4IV site in the proximal promoter region: DMRs on *Ntng1*, which was identified in this study, is an example of novel T-DMRs not described in the previous studies.

Several converging lines of evidence have indicated the significance of DNA methylation in normal brain function. Mutations in *Dnmt1*, *Dnmt3b*, and *Mecp2* result in functional and/or morphological abnormalities in human and mouse brain [26-28]. Mutations in the human orthologs of some genes carrying NSph-T-DMR, such as *LIG4* and *NFIA*, are associated with neurological disorders [29,30]. Similar phenotypes are observed in mice harboring mutations in these genes [4,31]. Targeted mutation of 2 genes with E11Hypo-T-DMRs, *Dclk1* and *Nrcam*, results in axonal defects in mice [32,33]. Disorganized DNA methylation profiles have been reported in cloned animals [34], chemically treated cells [35], and in certain diseases [36,37]. Epimutations in tumor suppressor genes are involved in carcinogenesis [38]. Therefore, the establishment of DNA methylation status at T-DMRs in these genes indicates the possibility that epimutations at T-DMRs could be involved in neurological disorders without genetic alterations.

## Conclusions

The dynamics of T-DMRs, several of which are often identified around TSS of a single gene during neural development, contribute regulation of developmental expression of genes and the DNA methylation profile of mouse adult brain. The identified T-DMRs could be used for evaluation and identification of NPCs, and for epimutation analysis in neural diseases.

## Methods

### Tissue samples, neurosphere culture, and immunocytochemistry

All experiments using mice were carried out according to the institutional guidelines for the care and use of laboratory animals (Graduate School of Agricultural and Life Sciences, the University of Tokyo). Pregnant C57BL/6 N mice were euthanized, and fetuses were recovered in ice-cold phosphate-buffered saline (PBS) containing 0.6% glucose. For neurosphere culture, dissected telencephalons were dispersed, and were suspended in progenitor cell culture medium: Dulbecco’s modified Eagle’s medium (DMEM)/F12 (1:1) containing 5.5 mM HEPES, 2 mM L-glutamine, 1/50 volume of B-27 Supplement (Invitrogen), 20 ng/ml epidermal growth factor (EGF), 20 ng/ml human basic fibroblast growth factor (bFGF) (PeproTech), and 5 μg/ml heparin. Cells were seeded into a petri dish and cultured for 6 days, replacing half of the medium with fresh medium at day 3. To induce differentiation, cells were dispersed, suspended in differentiation medium (progenitor cell culture medium without EGF, bFGF, and heparin), and seeded onto poly-L-lysine- and laminin-coated coverslips for immunocytochemistry. After 4 days of culture, cells were fixed with 4% paraformaldehyde and stained with a monoclonal antibody against βIII-tubulin (Covance) and a rabbit polyclonal antibody against glial fibrillary acidic protein (DAKO).

### Genomic DNA extraction

Cells (1.5 × 10^6^) were incubated in 200 μl of lysis solution (10 mM Tris–HCl (pH 8.0), 5 mM EDTA, 200 mM NaCl, 0.2% SDS and 200 μg/ml proteinase K) at 55°C for 30 min. The samples were extracted with phenol/chloroform/isoamyl alcohol (PCI; 25:24:1), incubated with RNase for 30 min, and extracted again with PCI. Genomic DNA was precipitated with ethanol and dissolved in 20 μl of TE (pH 8.0).

### D-REAM

D-REAM was performed as previously described [7]. Genomic DNA (5 μg) was digested with HpyCH4IV (New England Biolabs), extracted with PCI and chloroform, ethanol-precipitated, and dissolved in TE (pH 8.0). DNA sample (50 ng) was ligated to the R-adaptor pair using T4 DNA ligase (New England Biolabs) at 16°C overnight. After the 5^′^-overhang of the adaptor was filled in with Klenow Fragment, the DNA was digested with TaqI at 65°C for at least 1 h and purified with a Microspin S-300 HR Column (GE Healthcare). The TaqI ends of the DNA were ligated to the N-adaptor pair. The resulting DNA sample was purified with the Wizard SV Gel and PCR Clean-up System (Promega) and amplified with the R18 and N18 primers and Immolase Taq DNA polymerase (Bioline) under the following conditions: denaturation at 95°C for 7 min, followed by 20 cycles of 30 sec at 95°C, 30 sec at 62°C, and 2 min at 72°C. DNA was purified with MinElute PCR Purification Kit (Qiagen), and 7.5 μg of DNA was used for microarray analysis. Microarray analysis was conducted with GeneChip System (Affymetrix), and all procedures were done according to the Affymetrix Chromatin Immunoprecipitation Assay Protocol. DNA samples were labeled with the GeneChip WT Double-Stranded DNA Terminal Labeling Kit and hybridized with GeneChip Mouse Promoter 1.0R Arrays. Arrays were stained and washed with GeneChip Fluidics Station 450 and scanned with GeneChip Scanner 3000 7 G. The instruments were operated with GeneChip Operating Software version 1.4. D-REAM data obtained in this study have been deposited in the ArrayExpress database (accession number E-MTAB-1150). The D-REAM dataset of the adult whole cerebrums (AdBr), which were obtained from 13 week-old male mice, is reported previously [7].

### Data analysis

D-REAM data for two experiments were obtained for each NSph. The data were visualized using the Integrated Genome Browser (Affymetrix). Ensembl Transcript IDs (release 46) associated with T-DMR were obtained using BioMart [39] and Galaxy website [40]. Distribution analysis was conducted with the R software package. K-means clustering of MATscores was performed with the MultiExperiment Viewer (MeV in TM4 Microarray Software Suite) [41]. Gene Ontology analysis was conducted using the DAVID Bioinformatics Resources website [42].

### Combined bisulfite restriction analysis (COBRA) and sequencing

PstI- or EcoRV-digested genomic DNA (3 μg) was denatured with 0.3 M NaOH. Sodium metabisulfite (pH 5.0) and hydroquinone were added to final concentrations of 2.0 M and 0.5 mM, respectively. The reaction mixtures were incubated in the dark at 55°C for 16 h. The DNA was purified with the Wizard DNA Clean-up System (Promega), treated with 0.3 M NaOH at 37°C for 15 min, and ethanol-precipitated. The DNA was dissolved in 20 μl of TE (pH 8.0). After the bisulfite reaction, the unmethylated CpGs are converted to uracil-phosphate-guanines (UpGs), whereas the methylated CpGs remain intact. One-hundredth to 1/20 amount of the DNA was used for PCR with Immolase Taq DNA polymerase. For COBRA, one-tenth of the PCR product was digested with HpyCH4IV at 37°C overnight and electrophoresed with untreated control in a 2% agarose gel. For sequencing, PCR product was purified with the Wizard SV Gel and PCR Clean-up System (Promega) and cloned into pGEM-T Easy Vector (Promega). Up to 16 clones were sequenced. Primer sets used are listed in Additional file 5: Table S2.

### RT-PCR

Total RNA was extracted with the TRIzol Reagent (Invitrogen), and 1 μg of total RNA was subjected to reverse transcription using the Superscript II First-strand Synthesis System (Invitrogen). One-hundredth of the cDNA was used for PCR with Immolase Taq DNA polymerase under the following conditions: denaturation at 95°C for 7 min and 23 or 35 cycles of 30 sec at 95°C, 30 sec at 62°C, and 30 sec at 72°C (Additional file 5: Table S2). Quantitative RT-PCR was carried-out on Bio-Mark HD system (Fluidigm) using Universal probes (Roche Applied Science) for monitoring amplifications (detailed in Additional file 6). Makers were selected according to the previous report [43].

## Abbreviations

T-DMRs: Tissue-dependent and differentially methylated regions; TSSs: Transcription start sites; AdBr: Adult brain; CGIs: CpG islands; NPCs: Neural progenitor cells; NSph: Neurospheres; D-REAM: T-DMR profiling with restriction tag-mediated amplification; COBRA: Combined bisulfite restriction analysis; GO: Gene ontology; Q-RT-PCR: Quantitative reverse-transcription polymerase chain reaction.

## Competing interests

The authors declare no conflicts of interest.

## Authors’ contributions

KH, KS and SY designed this study. KH and SY performed the experiments, and analyzed data. KH, SY and KS prepared the manuscript. All authors read and approved the final manuscript.

## Supplementary Material

Additional file 1: Figure S1 NSph differentiation capacity. Click here for file

Additional file 2: Table S1 Associations of genes carrying NSph-T-DMRs with human neurological disease. Click here for file

Additional file 3: Figure S2 Integrated Genome Browser (IGB) images of the genes (Ensembl Transcripts) with E11Hypo- (A) and E14Hypo-T-DMRs (B). Click here for file

Additional file 4: Figure S3 COBRA representing DNA methylation status of NSph-T-DMRs. Click here for file

Additional file 5: Table S2 Primers used in this study. Click here for file

Additional file 6 Method and Primer list for Q-RT-PCR. Click here for file
