# Supplementary material for: DNA methylation profile dynamics of tissue-dependent and differentially methylated regions during mouse brain development
Source: BMC Genomics. 2013 Feb 6;14:82. doi: 10.1186/1471-2164-14-82 (PMC3599493; doi:10.1186/1471-2164-14-82)
Supplement: Additional file 1: Figure S1 — NSph differentiation capacity. [file 1471-2164-14-82-S1.pdf]

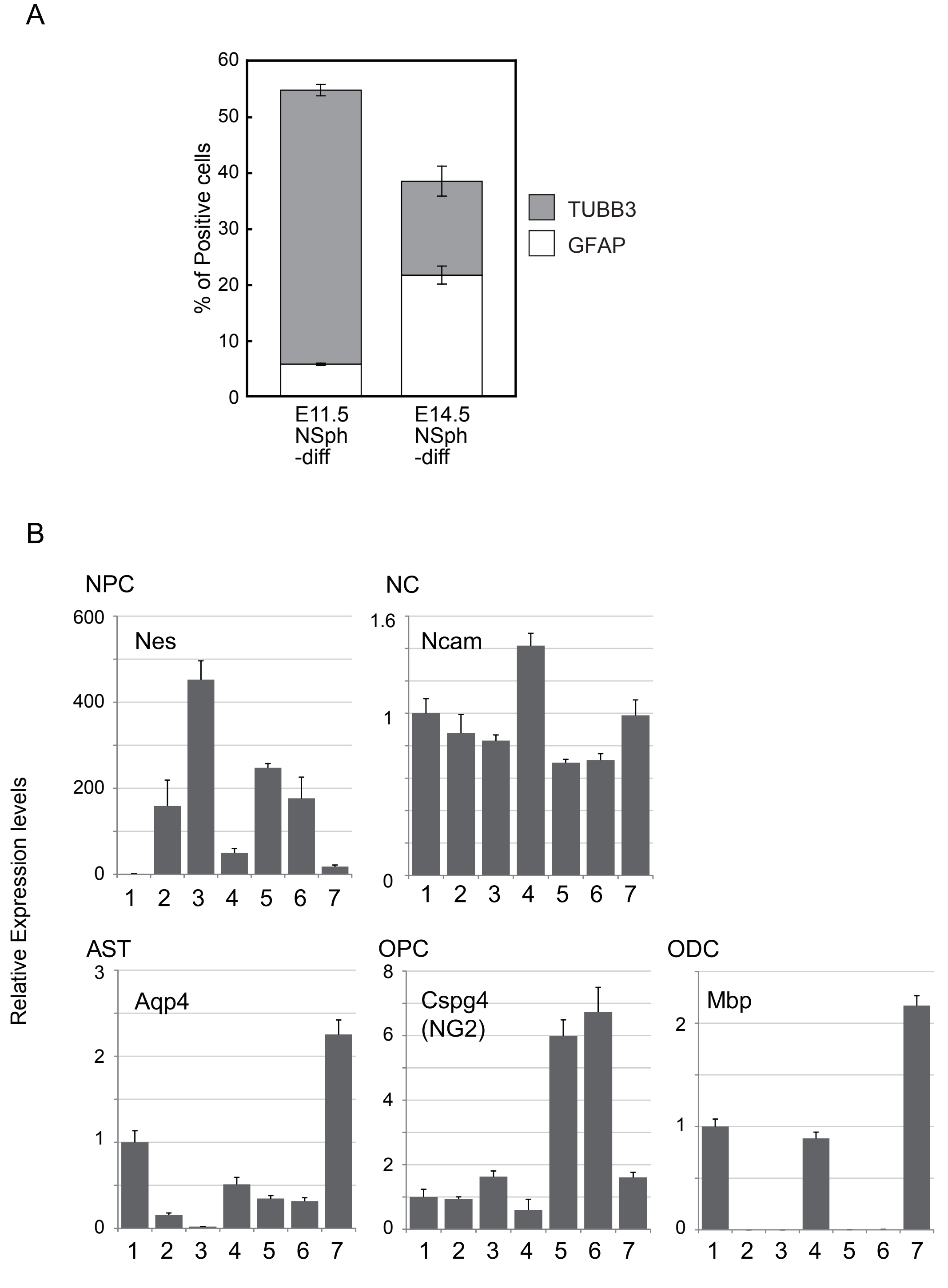

Figure S1. Expression of marker genes for neural cell lineage.

A: Immunocytochemical staining of differentiated E11.5NSph (E11.5NSph-diff) and E14.5NSph (E14.5NSph-diff) was conducted using antibodies against TUBB3 and GFAP. Percentages of TUBB3-positive and GFAP-positive cells in DAPI-positive cells were counted and are presented as mean ± SE of 3 visual fields.

B: Quantitative RT-PCR for marker genes. Expression levels of marker genes—nestin (Nes): neural progenitor cells (NPC); neural cell adhesion molecule (Ncam): neuronal cells (NC); aquaporin 4 (Aqp4): astrocytes (AST); NG2 (Cspg4): oligodendrocyte progenitor cells (OPC); myelin basic protein (Mbp): oligodendrocytes (ODC)— were normalized with the expression levels of β-actin (Actb) and indicated by relative expression levels to those of adult brain (AdBr). Error bars indicate SE of tripricated wells of a sample (1: AdBr; 2, 3: E11.5 NSphs; 4: differentiated E11.5 NSphs; 5, 6: E14.5 NSphs; 7: differentiated E14.5 NSphs).
