# Supplementary material for: DNA methylation profile dynamics of tissue-dependent and differentially methylated regions during mouse brain development
Source: BMC Genomics. 2013 Feb 6;14:82. doi: 10.1186/1471-2164-14-82 (PMC3599493; doi:10.1186/1471-2164-14-82)
Supplement: Additional file 3: Figure S2 — Integrated Genome Browser (IGB) images of the genes (Ensembl Transcripts) with E11Hypo- (A) and E14Hypo-T-DMRs (B). [file 1471-2164-14-82-S3.pdf]

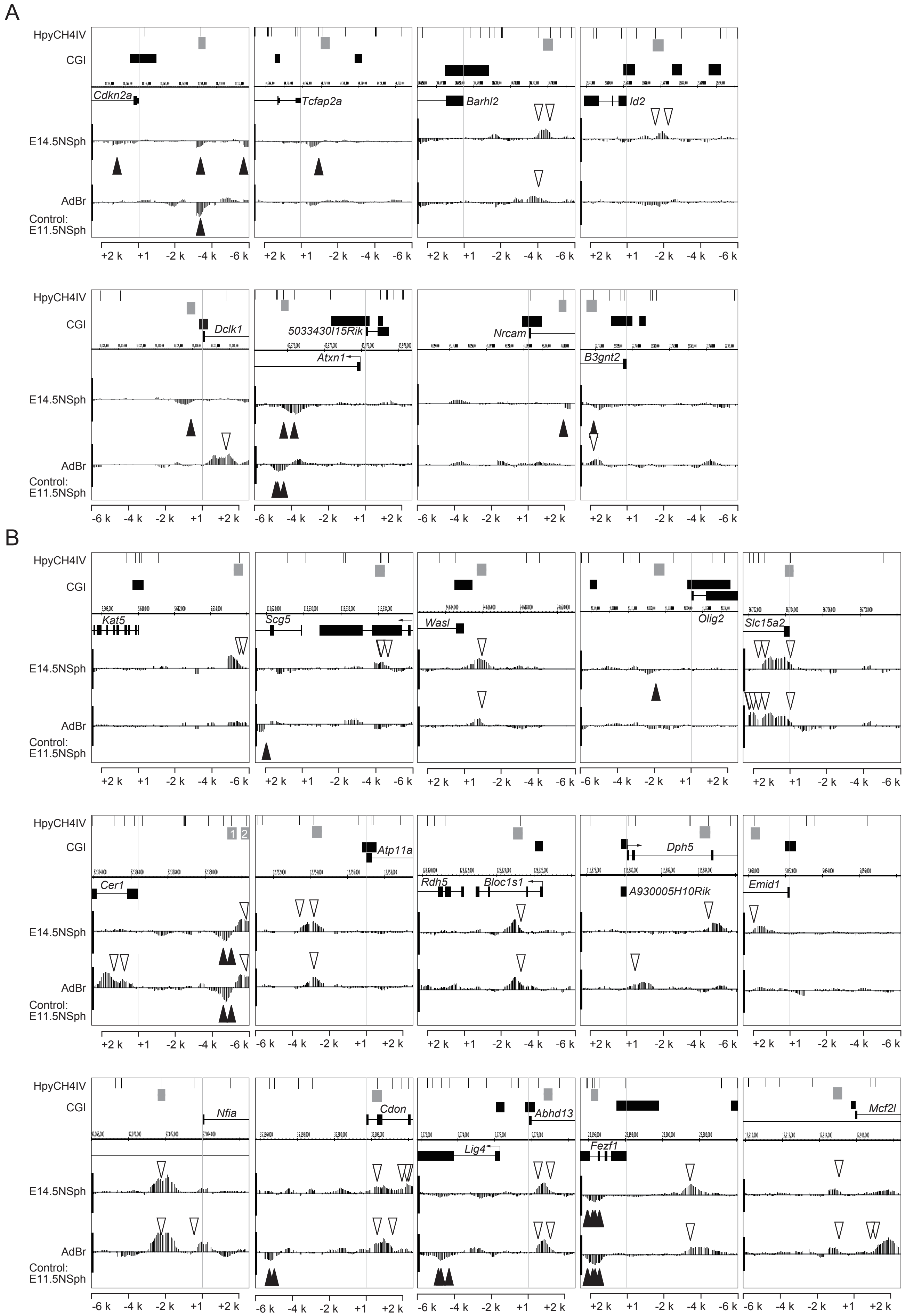

Figure S2. Positions of T-DMRs identified by D-REAM analysis

Panels indicate Integrated Genome Browser (IGB) images for the genes (Ensembl Transcripts) with E11Hypo- (A) and E14Hypo-T-DMRs (B). Comparative MATscores of E14.5NSph and the adult brain (AdBr) to E11.5NSph as the control were plotted and displayed from 6 kb upstream to 2.5 kb downstream of the transcription start site (TSS) at the middle parts of each panel. Filled and open arrowheads indicate T-DMRs hypermethylated and hypomethylated in comparison to E11.5NSph, respectively. Regions analyzed by bisulfite sequencing or COBRA are indicated by gray rectangles.
