# Supplementary material for: DNA methylation profile dynamics of tissue-dependent and differentially methylated regions during mouse brain development
Source: BMC Genomics. 2013 Feb 6;14:82. doi: 10.1186/1471-2164-14-82 (PMC3599493; doi:10.1186/1471-2164-14-82)
Supplement: Additional file 4: Figure S3 — COBRA representing DNA methylation status of NSph-T-DMRs. [file 1471-2164-14-82-S4.pdf]

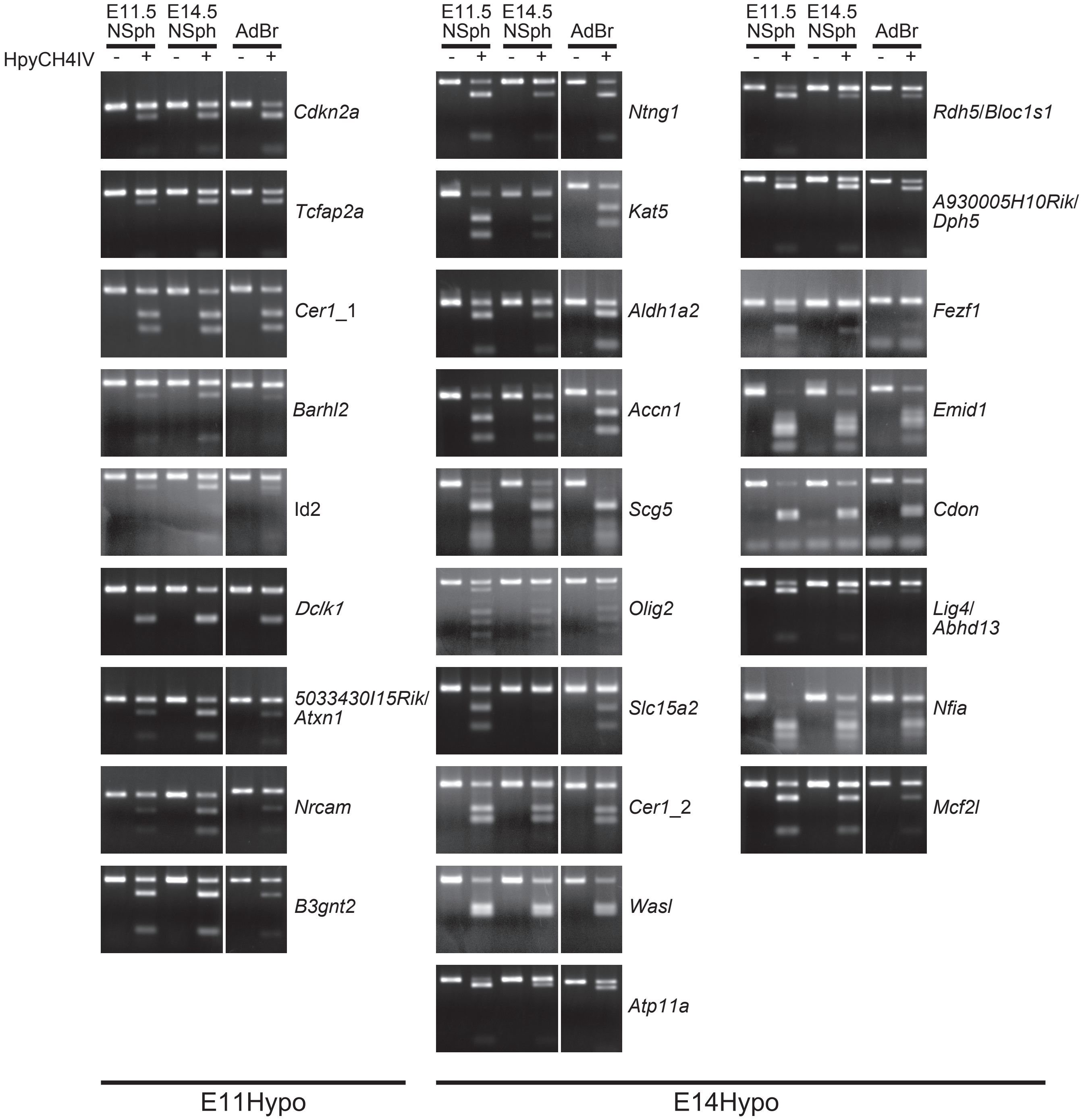

Figure S3. COBRA for NSph-T-DMRs

Agarose EP gel images for COBRA of NSph-T-DMRs are indicated. Bisulfite PCR products were electrophoresed after incubation without (-) or with the restriction enzyme, HpyCH4IV (+). The target regions are indicated by gray rectangles in Figure S2. Primer sets used are listed in Table S2.
