# Supplementary material for: DNA methylation profile dynamics of tissue-dependent and differentially methylated regions during mouse brain development
Source: BMC Genomics. 2013 Feb 6;14:82. doi: 10.1186/1471-2164-14-82 (PMC3599493; doi:10.1186/1471-2164-14-82)
Supplement: Additional file 6 — Method and Primer list for Q-RT-PCR. [file 1471-2164-14-82-S6.doc]

**Quantitative RT-PCR.** cDNA samples, equivalent to 25 ng RNA, were pre-amplified using TaqMan PreAmp Mastermix (Roche Applied Science) according to manufacturer's protocol. Following pre-amplification, the samples were diluted 1:5 with sterilized H_2_O. Primers and Universal probes (listed in below table) were purchased from Sigma-Aldorich and Roche Applied Science, respectively. Following loading of the probes and samples into a BioMark 48.48 dynamic array (Fluidigm), PCR was performed with the following reactions conditions: 50°C for 2 min, 95°C for 10 min, followed by 40 cycles of 95°C for 15 sec and 60°C for 45 sec. Fluorescent signals, which were captured at the end of each cycle, were processed using BioMark Real-time PCR Analysis software. Primers were designed on Universal ProbeLibrary Assay Design Center (www.universalprobelibrary.com).

| Gene Symbol | Primer | Sequence | Universal Probe |
| --- | --- | --- | --- |
| *Ntng1* | mNtng1_L | CAGGGGCAAGAGACCAAG | #103 |
|  | mNtng1_R | AGGGATGGTGTCTATCGTCCT |  |
| *Nes* | Nes_taq_F | CTGCAGGCCACTGAAAAGTT | #1 |
|  | Nes_taq_R | TCTGACTCTGTAGACCCTGCTTC |  |
| *Ncam* | Ncam_taq_F | AGGGCAAGGCTGCTTTCT | #69 |
|  | Ncam_taq_R | CCCCATCATGGTTTGGAGT |  |
| *Aqp4* | mAqp4_L | TGGAGGATTGGGAGTCACC | #22 |
|  | mAqp4_R | TGAACACCAACTGGAAAGTGA |  |
| *Cspg4* | mCspg4_L | CAGAAGGGACCAGCTAGAGG | #16 |
|  | mCspg4_R | GGGCTTCTCAACGAGAACAT |  |
| *Mbp* | mMbp_L | GCACGCTTTCCAAAATCTTTA | #69 |
|  | mMbp_R | GCCATGGGAGATCCAGAG |  |
| *Actb* | Actb_taq_F | GGATGCAGAAGGAGATTACTGC | #63 |
|  | Actb_taq_R | CCACCGATCCACACAGAGTA |  |
